# Highlighting the Structure-Function Relationship of the Brain with the Ising Model and Graph Theory

**DOI:** 10.1155/2014/237898

**Published:** 2014-09-04

**Authors:** T. K. Das, P. M. Abeyasinghe, J. S. Crone, A. Sosnowski, S. Laureys, A. M. Owen, A. Soddu

**Affiliations:** ^1^Physics & Astronomy Department, Brain & Mind Institute, Western University, London, ON, Canada N6A 3K7; ^2^Neuroscience Institute & Centre for Neurocognitive Research, Christian Doppler Klinik, Paracelsus Medical University, 5020 Salzburg, Austria; ^3^Centre for Neurocognitive Research & Department of Psychology, University of Salzburg, 5020 Salzburg, Austria; ^4^Department of Neurology, Christian Doppler Klinik, Paracelsus Medical University, 5020 Salzburg, Austria; ^5^Cyclotron Research Center and University Hospital of Liège, University of Liège, 4000 Liège, Belgium; ^6^Department of Neurology, CHU Sart Tilman Hospital, University of Liège, 4000 Liège, Belgium; ^7^Department of Psychology, Brain & Mind Institute, Western University, London, ON, Canada N6A 5B7

## Abstract

With the advent of neuroimaging techniques, it becomes feasible to explore the structure-function relationships in the brain. When the brain is not involved in any cognitive task or stimulated by any external output, it preserves important activities which follow well-defined spatial distribution patterns. Understanding the self-organization of the brain from its anatomical structure, it has been recently suggested to model the observed functional pattern from the structure of white matter fiber bundles. Different models which study synchronization (e.g., the Kuramoto model) or global dynamics (e.g., the Ising model) have shown success in capturing fundamental properties of the brain. In particular, these models can explain the competition between modularity and specialization and the need for integration in the brain. Graphing the functional and structural brain organization supports the model and can also highlight the strategy used to process and organize large amount of information traveling between the different modules. How the flow of information can be prevented or partially destroyed in pathological states, like in severe brain injured patients with disorders of consciousness or by pharmacological induction like in anaesthesia, will also help us to better understand how global or integrated behavior can emerge from local and modular interactions.

## 1. Introduction

Despite decades of research focusing on network based brain activities, the anatomical structure of the observed brain networks and the functional aspects of spatiotemporal brain dynamics remain mysterious [1]. Several recent functional magnetic resonance imaging (fMRI) studies on the wakeful resting brain have showed the existence of different brain networks—resting-state networks (RSNs), for example, the default mode network (DMN), salience network (SN), auditory network (AN), three distinct visual networks (VN), sensorimotor network (SMN), and left and right executive control (ECN), which are considered to be unperturbed, nonstimulated functional networks, which at baseline activity are performing complex cognitive tasks [2–6]. The balance between segregation and integration of well-segmented and separated brain regions is essential for efficient information processing and rapid information transfer within and between the networks [7, 8]. A human brain consists of around 100 billion neurons, and each of these neurons forms approximately 1000 trillion electrical as well as chemical synaptic and nonsynaptic connections with other neurons in a brain network [9]. As a result, the importance of studying neurobiological implications of the complex neural wiring structure of these aforementioned functional networks has always been proven to be critical. The state of the art in neuroimaging techniques is already offering us the possibility to assess structural and functional connectivity of these brain networks. However, there is still a gap in finding more convincing structure-function relationships that could be predicted by the optimal neural activity in the network. This limits our current understanding of the mechanisms governing the base of emergent spatiotemporal brain dynamics, and their relation to complex evolutionary cognitive assessments of brain networks [10].

**Figure 1:**
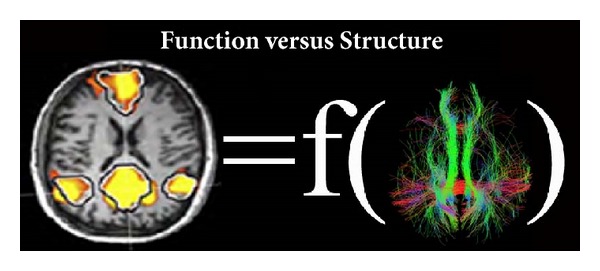
Default mode network in a healthy control as extracted from resting state functional magnetic resonance imaging using independent component analysis and the fibers reconstructed using a tractography technique applied to diffusion tensor imaging data and subsequently filtered by the regions functionally connected in the default mode network.

Starting from the Hodgkin-Huxley conductance-based model [11], the field of computational neuroscience has been playing a significant role in replicating the functional characteristics of spontaneous neuronal activity from the modular brain network [12–16]. This could provide new insights into network dynamics along with the advancement of neuroimaging experiments [17–20]. Neuroimaging observations are strongly supporting a relationship between the structural architecture of the brain and its functional networking (see Figure 1 as an example for the DMN functional pattern and its structural support) [21–23]. The emergence of spontaneous network dynamics in the resting brain was simulated from the heterogeneous structural connectivity of the human brain and then compared with the spatiotemporal dynamics of BOLD low-frequency signals during rest [19]. In their study, oscillatory neural populations were found to be synchronized through the phase dynamics of coupled oscillators in a generalized Kuramoto model. This synchronization model could offer a detailed comparison of functional networks among the structural connectivity. With increasing global coupling strength of oscillators, clusters of oscillator nodes progressively integrate to form larger clusters with positive and negative correlations between them, and the corresponding network dynamics exhibit a phase transition from a desynchronized phase to a partially synchronized phase. In contrast to homogeneous or randomly coupled networks, the transition from a partially synchronized to a fully synchronized phase was found to be less probable due to the role of possible delay in transmission in the resting brain network. Despite this, a good agreement of “emergent” properties between simulated functional connectivity and empirical functional connectivity was demonstrated through the measurement of individual seed-based correlation in RSNs. Mechanisms behind cluster formations (or integration), intra- and inter-regional interactions, and the emergence of intercluster correlations/anticorrelations remain unexplored [19, 22]. Their studies suggest the need for more computational modeling-based research on the structure-function dependencies in brain networks.

In the context of the complex structure-function relationships in the brain network, self-organized neural dynamics have been shown to exhibit random behaviour which can become very similar to systems studied in statistical physics (e.g., the Ising model) [24–26]. In the past, neural dynamics in the resting brain had been considered as quasistationary states without introducing the structural information and without a direct correspondence between lattice size and brain positions [12, 27]. In order to investigate dynamics of the resting brain, a collective phenomenon based 2-dimensional (2D) Ising model was simulated numerically at different temperatures [18, 28]. Their simulated results allow an assessment of the cooperative emergent properties and the universality classes of network dynamics [18, 20, 27] as well as the biological plausibility of RSNs [2]. Under these mechanisms, the collective spin dynamics exhibit long-range spatiotemporal correlations with second order phase transitions between ordered and disordered magnetic states at the critical temperature [12, 29]. Compared to subcritical (*T* < *T*_*c*_) and supercritical (*T* > *T*_*c*_) temperature regions, their simulated results at this critical point highlighted a balance between positive and negative correlated networks and were comparable with the correlation and anticorrelation obtained from resting state fMRI. The universal mechanisms underlying the spontaneous emergent phenomena of the 2D Ising model can explain self-organized criticality of neural dynamics in large scale RSNs [28, 30]. Functional activation patterns of neural networks largely depend on the underlying structure of fiber pathways connecting all regions of the cortical and subcortical brain area [23, 31]. In a recent study [32], the functional neural activity of resting brain networks was also simulated from the generalized Ising model, replacing equal spin coupling with the structural network of the human connectome. The structural based collective neural dynamics were able to explain correlation-based networks that were comparable with the RSNs extracted from fMRI. Their findings confirmed that the second order phase transition and self-organized criticality of the 2D Ising model at critical temperature [27] might not be enough to explain the complex organization of information transfer in resting brain networks. This raises more puzzling questions about functional RSNs in various conditions including the involvement of cognitive tasks on the resting brain or altered states of consciousness like physiological (sleep), pharmacological (anesthesia), and pathophysiological (disorder of consciousness) states of brain networks [33].

In addition to the aforementioned Kuramoto model of coupled oscillators and Ising model of magnetization, Honey et al. presented a neuronal mass model based analysis that could predict the functional connectivity from the human anatomical structure [21]. Following conductance-based neuronal dynamics, the neural mass model simulates a population of highly interconnected excitatory and inhibitory neurons. Three dynamic variables (membrane potential of pyramidal cells, average number of open potassium channels, and inhibitory interneurons) are used to describe the time-course of local field potentials for a neuron mass. Each mass represents a node of cortex, which is interconnected to other nodes through the structural connectivity matrix via the mean firing rate. Along with these variables there are multiple microscopic quantities (ion channel conductance, fraction of channels open, and the ratio of NMDA to AMPA receptors), being used to describe the dynamics of neural masses. The number of intraconnected neurons, as well as the net effect of these microscopic constants, remain unknown for each node. There is an additional fitting parameter describing the excitatory coupling between nodes in the network. Utilizing all parameters, global resting brain dynamics are modelled and compared with empirical results [21, 34, 35]. In comparison, the Ising model uses one parameter, temperature, to simulate global brain activity. More research on the Ising model, taking into account the structural and functional interrelationships seen in the neural mass model, could resolve many unknowns in large-scale brain networks.

Modern neuroimaging techniques like fMRI and diffusion tensor imaging (DTI), along with methodological advances in both spatial pattern detection and anatomical tracing, has made it possible to extract the functional patterns and the structures of neuroanatomical circuitry at different spatial scales [36–38]. With the development of graph theory, we have witnessed an unprecedented growth of applications to understand the structural and functional complexity of the human brain connectome [39] due to its relative simplicity, highly generalized, and easily interpretable nature. In a graph, structural (i.e., synaptic, axonal, and dendritic) and functional (i.e., spontaneous or evoked neuronal response based dynamic interactions) network connectivity of brains is typically represented by a set of nodes which carries neuronal information at the scale of interest and a set of edges that represents either functional relationships or structural connections among individual nodes [10, 40, 41]. Correlated nodes in patches of the cortex (gray matter) were used to demonstrate dynamic interaction of neural circuitry, in which functionally clustered regions of small-world networks were governed by specific features, for example, high clustering, small path length, high efficiency, and repeated network motifs in a particular class [42, 43]. In the Watts-Strogatz model, probability of increasing rewiring demonstrates the transition of a random network from a periodic ring shaped lattice of the small-world topology [44]. Several pathological states of brain networks were also investigated, in which any disturbance of structural connectivity in neural networks could increase the probability of rewiring and reduce the functionally organized brain activity, for example, the Erdös-Rényi type networking [34, 45–47]. Based on anatomical connectivity patterns and physiological interactions of neurons in mammalian brains, a statistical model of canonical microcircuits was able to describe cortical dynamics dependent on the large-scale “average connectivity” [14, 48]. The linked long-range projections in this model demonstrated nonrandom coherent features and large-scale spatiotemporal organizations of complex brain functionality. Recently, an electroencephalogram (EEG) study providing a high temporal resolution has been performed on large-scale network dynamics to investigate the loss of consciousness and cognitive deficits in patients with disorders of consciousness (DOC) after severe brain injury. This study provides information about further diagnosis and physiological mechanisms [49]. In large-scale brain networks, however, nodes keep being defined a priori, which is not always justified due to the limitation in sensitivity of detecting complex axonal fiber architecture [49, 50] and also due to the lack of appropriate parcellation procedures in order to establish short- and long-range functional relationships among highly coherent brain regions [51, 52].

The performance of self-organized criticality, and its relation to efficient information processing in conscious brains, is solely determined by maintaining an optimal balance between axonal lengths and synaptic costs in neuronal circuitry [53]. Axonal wiring cost is considered as the source of functional integration, which is mostly spent forming long-range communications among spatially distant brain regions. On the other hand, the strength of short-ranged intraregional connectivity is improved with an increase of synaptic costs, implying a segregation effect on global dynamic patterns. There are also other factors, for example, the metabolic cost, glia cells, and myelination that play a role in neuronal communications. According to the economic principle of the brain, minimizing wiring and metabolic energy costs results in a more “profitable” and efficient tradeoff between wiring costs and the maximum structural and/or functional connectivity among spatially distinct brain regions. Balancing neuronal communication cost and highly conserved global connectome organization, the functional network topology in the healthy human brain demonstrates small-worldness [45]. The computational efficiency and functional integration of this type of brain network lie in the intermediate regime between the lattice-type topological networking (efficiency = low, cost = low) and random networking (efficiency = high, cost = high) [54]. Series of earlier fMRI studies on the anaesthetized human and monkey brains demonstrated a breakdown of cortical and subcortical functional connectivity in all resting networks when subjects were in a state of anesthesia-induced loss of consciousness [55–58]. This effect on functional connectivity altered intra- and inter-cortical connectivity, preventing the efficiency of information flow that was present in the small-world network of wakeful healthy brains. Their findings on imbalanced functional connectivity in the segmented cortical network also highlighted the requirement of maintaining the economic principle in order to perform the active cross-modal functional interactions during network communications.

In this paper, we focus on reviewing the functional organization of brain dynamics and its underlying structure-function relationship in a wakeful and conscious resting brain, followed by a brief discussion of its alterations under pharmacological and pathological states of consciousness [33]. Recent work on the Ising model and graph theory is explored to help understanding the global and local organization of brain communication at a spatiotemporal scale [20, 32, 59, 60] and its structural-functional interdependencies. Comparing both theoretical insights and fMRI empirical results, the notion of criticality, metastability, and phase transitions in self-organized brain dynamics are demonstrated, taking into account the emergence of macrostates under cooperative processes [13, 61].

## 2. Ising Model and Its Application to Brain Dynamics

**Figure 2:**
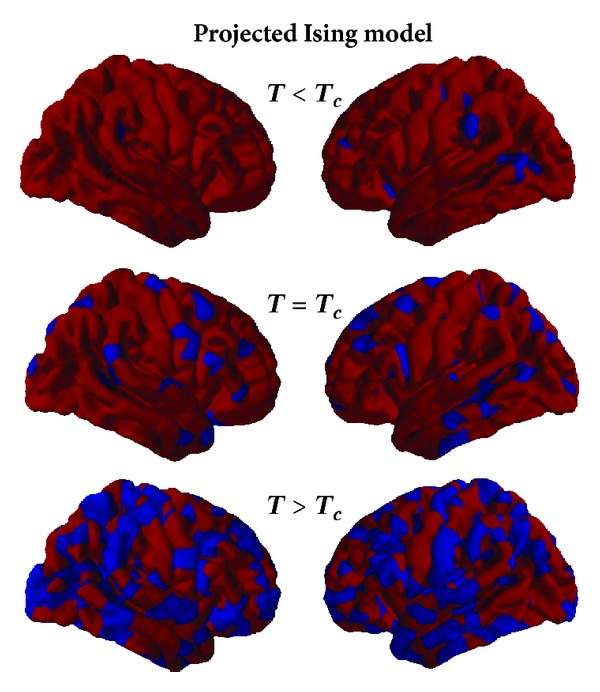
Three different 2D Ising model configurations after thermalization for, respectively, *T* < *T*_*c*_, *T* = *T*_*c*_, and *T* > *T*_*c*_. A 32 × 32 square lattice configuration has been projected on a 1015 parcellated brain keeping the nearest neighbour interaction structure.

The Ising model of ferromagnetism was firstly introduced by Ernst Ising in 1925 as a statistical model of ferromagnetism [62]. A 2D square lattice version was further explored to explain the existence of ferro/paramagnetic transitions and was exactly solved in statistical physics by Onsager in 1944 [63]. Essentially, the model consists of discrete magnetic moments with their spins *s*_*i*_ = +1 pointing upward and *s*_*i*_ = −1 pointing in downward directions. In the brain, local increase (or decrease) in BOLD activity from its baseline could also be represented by “+1” (or “−1”) spin state [18, 64]. According to the model, each of these spins has the tendency to align with its neighbours in the square lattice through the nearest-neighbour, interaction energy, or coupling constant *J*_*ij*_. In the absence of any external field, the energy of a state *t* is expressed by *E*(*t*) = − (1/2)∑_〈*i*,*j*〉_ *J*_*ij*_ *s*_*i*_ *s*_*j*_, where 〈*i*, *j*〉 denotes the nearest neighbour interaction between nodes *i* and *j*. Furthermore, the strength of interactions always competes against the effect of the temperature of the thermal bath with which the spin lattice is in contact. According to the formalism of statistical physics [65], the probability of finding the system in a configuration *t* is *P*(*t*) = exp⁡⁡[−*E*(*t*)/*kT*]/*z*. Here *z* = ∑*e* ^−*E*(*t*)/*kT*^ is called the partition function, *k* is the Boltzman constant, and *T* is the temperature. *E* measures the integrated energy of a spin configuration, in which the interaction between spins wants to minimize which can be used to quantify the global spin organization. *kT* instead measures the kinetic energy randomly transferred between the thermal bath and the spin lattice, which produces the segregation effect in spin clusters. Numerical approaches have been developed to simulate the dynamics of an Ising model. The combination of Metropolis algorithm and classical Monte Carlo simulation with periodic boundary conditions establishes the thermalization procedure of magnetic spins when the system is placed in touch with a heat bath of temperature *T* [66]. With increasing *T*, the spontaneous fluctuation of Ising spins increases. When the temperature reaches a certain critical value (*T*_*c*_), there is a qualitative change in the organization of the spin clusters as a whole, and the correlation length between neighbouring spins within the cluster (which captures the size of the formed clusters) diverges. The outcome of spin organizations from a 2D Ising model simulation at three different *T* values is presented in Figure 2 after being projected on a 1015 parcellation of the brain [67] (notice that the relation between physical position in the brain and the 2D lattice is completely arbitrary, even if nearest neighbour interaction distribution is maintained).

**Figure 3:**
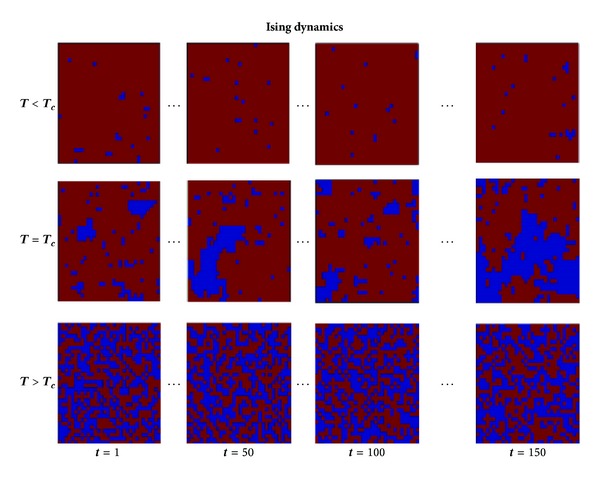
Dynamics of a 2D Ising model with lattice size 32 × 32 after thermalization. For the three different temperatures *T* < *T*_*c*_, *T* = *T*_*c*_, and *T* > *T*_*c*_ the Ising model is simulated generating 150 time data points. Each time point corresponds to a new configuration in which all spins have been tested for flip through the Metropolis algorithm. Configurations of the same four time points are reported for the three different temperatures.

At low *T*, the spontaneous Monte Carlo spin flips are less probable, and spins in a given configuration are mostly aligned to contribute the minimum energy or ground state energy of the system. A quantity called the magnetization, which is the average of total spins over the whole lattice, determines the magnetic ordering of the system, that is, its ferromagnetic behaviour. When all spins are aligned along the same direction, a magnetization of magnitude “+1” or “−1” will be generated corresponding to a complete order configuration. The large amount of integrated magnetic ordering in this low temperature regime is accompanied by a small information content of the organized spin clusters. At high *T*, the magnetic ordering is completely lost due to significantly increasing number of spontaneous spin flips and the magnetization tends to “0,” which can be used to characterize the paramagnetic phase. In this case, a large number of laws of nature do not hold due to spontaneous symmetry breaking under global spin flips, and this disordered phase can be seen as the result of the dominating segregation effect over the cluster integration [66]. The information content is very high, in this case, but without integration. For the intermediate regime of *T*, the self-organized criticality, as well as a second order phase transition, are observed in the 2D Ising model through the maximum fluctuation in the magnetization and the susceptibility peak when *T* reaches a critical value *T*_*c*_ [18]. In this case, a balance between integration and segregation effects is recognized and revealed by the divergence of the correlation length through the formation of long-range ordering within the correlated functional networks of spin clusters. The global ordering of magnetization is preserved in the *T* < *T*_*c*_ regime and is destroyed above the critical temperature *T*_*c*_. In Figure 3, four different states of 2D spin configurations are shown for *T* < *T*_*c*_, *T* = *T*_*c*_, and *T* > *T*_*c*_. These four different configurations correspond to the same four different time points at three different temperatures. Due to its simplicity of simulating two state spin systems and the richness of its dynamic behaviour in self-organized criticality, the Ising model has been demonstrating unprecedented growth of applications in physics as in many other fields, including computational neuroscience [18, 28, 30].

**Figure 4:**
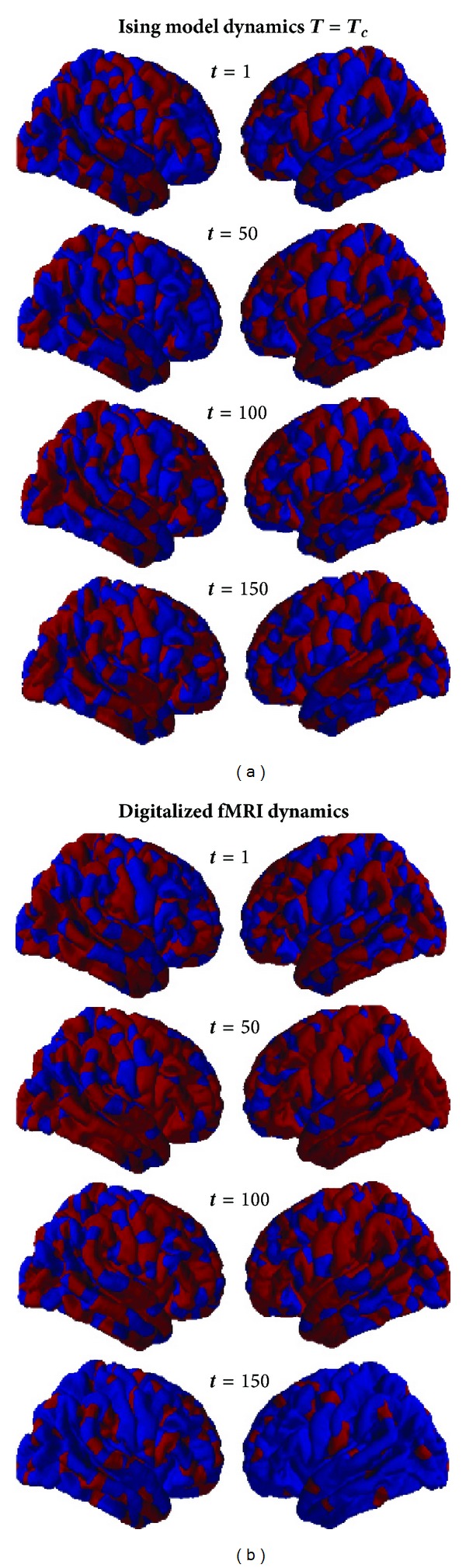
Simulated and functional imaging maps generated for four different time points. (a) shows the equilibrium spin configurations at *T* = *T*_*c*_. In (b), digital maps are created by setting a threshold as the baseline value of the BOLD time course for each corresponding parcellated region. Red corresponds to a value of above baseline (or spin “+1”) and blue below (or spin “−1”). Maps are created after averaging BOLD signal over 14 healthy subjects.

In neuroscience, electrophysiological brain activity in the presence or absence of sensory stimulation can be described by two states: (1) active states in which randomly generated neuronal action potentials collectively process information and provide neuronal communications with each other via functional networking and (2) inactive states in which neurons do not cross the threshold value to fire action potentials [12]. In the brain at rest, when a large number of neurons are functionally connected with each other, the resultant interaction of all other neurons on a given neuron can always be considered as its single averaged form [68, 69]. This situation can often be realized in the mean field theory, in which an effective interaction (e.g., exchange coupling in the Ising model) substitutes the many body interactions, involving the long-range ordering in the functional network [64, 70]. Reduction of many degrees of freedom in neural dynamics can therefore be simplified in an asymptotic form that results as the emergence of activated functional patterns [71]. The stability of these synchronous dynamic patterns in a network represents a neuronal firing state based on cooperative activity. In addition to four different spin configurations at the critical temperature (*T*_*c*_), mean functional organizations of neural dynamics, sampled at four different times and based on the resting state fMRI of 14 healthy subjects is shown in Figure 4. The baseline for the fMRI signal has been separately calculated for each parcellated region as the mean of the time-course for that region. All values above (or below) the baseline are represented in “Red” (or “Blue”).

Series of earlier studies on fMRI, multielectrode local field potential (LFP), and magnetoencephalography (MEG) [72] profoundly highlighted the spontaneous emergence of cortical and sub-cortical resting brain activity in human and non-human primates [73]. In their analyses, collective functional organization of RSNs were found to be very similar to the emergence of simulated organizations poised in 2D Ising model near the critical temperature (see previous section). Along with the simulated spatiotemporal brain activity near or at the critical point, brain functionality in RSNs encountered the maximization of information processing, taking into account the input sensitivity and dynamic range of activity patterns [26, 73]. Besides prominent matching of the long-range correlations in large-scale cortical networks, power law behaviour with a slope value −3/2 (represents the fractal dimension) and neuronal avalanches in small-scale networks were indicated in empirical and simulated data of the resting brain [18, 74].

In addition to this earlier work, there has been considerable growing attention on simulating the brain dynamics and its relation to self-organized criticality using the structure of human connectome from the DTI based measurements [32]. The fiber distributions between each pair of cortex parcellated regions could be in fact used as the input for the coupling *J*_*ij*_ between spin *i* and spin *j* in a generalized Ising model. In this way, all nodes including left and right hemispheres are interacting with each other, implying that any spin sees all the other spins as nearest neighbor even if with different couplings. Recently, a study on simulating the resting functional activity in monkeys and humans [73, 75] attempted to emphasize the finite size, scaling, and universality of brain dynamics. Along with the measurement of maximum information processing at criticality, their calculations on *T*_*c*_ (not in line with earlier findings) as well as critical exponents of magnetization, specific heat and susceptibility, could explore collective brain activities in different spatial scales [75]. In the next section, functional organizations of spontaneous brain activity will be reviewed in the light of dynamic phase transitions, while the phenomena of self-organized criticality and metastability will help to characterize the similar behaviour of organized activity patterns observed in the empirical data [72, 76].

## 3. Self-Organized Criticality, Phase Transition, and Metastability in Brain Networks

Criticality in any dynamic system, including the brain, can be characterized by a threshold that describes the boundary of phase transition between ordered and disordered patterns. In order to understand experimental findings of human functional brain activity, for example, the resting state fMRI, a large number of interacting spin systems has been modeled successfully as self-organized criticality [77]. Recently, the correlation networks of resting state fMRI data were compared with the correlation matrix of a 2D Ising model at different temperatures, in which spins were connected with the short ranged nearest-neighbour interactions [28]. In the Ising model, the self-organized dynamic patterns are formed through the spontaneous fluctuation of random spins, reducing degrees of freedom through non-linear interactions among functional units of spin clusters [65]. These functional units are characterized by reduced degrees of freedom and are represented by order parameters (e.g., the magnetization) [66]. With increasing *T*, the spontaneous fluctuation of spin-flips increases, and at critical temperature the dynamic phase transition replicates the long-range ordering in the spin dynamics. This effect of long-range ordering can provide the maximum information flow, which is reduced down abruptly either in the phase of ordered (*T* < *T*_*c*_ in subcritical phase) or completely disordered (*T* > *T*_*c*_ in supercritical phase) spin states. This has been considered as the self-organized criticality of a 2D Ising model, in which the maximum occurrence of metastable states [76] can mimic almost all fascinating properties in the wakeful resting brain. Self-organization in the resting brain is also the result of spontaneous neural dynamics that have shown features such as metastability in order to explain the efficient information processing in the network. It is only in the critical regime of the Ising model that we can retain these properties and simulate brain functionality effectively [13, 20, 73].

Starting from Turing instabilities in dynamical systems (1950), it has been shown that macrostates of brain wave oscillations can be formed out of cooperative processes, instabilities, rapid transitions between coherent states, pattern formations, and so forth [13, 78]. Due to the variability of synaptic couplings among large groups of neurons in an input-output based brain network, the ability to process and transfer information depends solely on integrating several functional counter-parts of the neural circuits of cortical and subcortical structures [79]. Taking into account the cooperation and competition in spontaneous neuronal oscillations, the basis of conscious brain activity lies in the state of metastability [80]. The dynamic states of brain oscillations lie in far-from-equilibrium regions, but it stabilizes over a long time period, explaining the ability to perform brain activities out of many random inputs from the external world [81]. The emergence of simulated spin dynamics in the Ising model and brain dynamics in level of consciousness, exhibit features of dynamic transitions between metastable states [18, 76]. Due to the limitation on appropriate fiber-tract modeling, the structural connectivity used in current neuroscience research seemed to overlook long-range projections and the polarization of fiber tracts. Insufficient information on the anatomical structure of the brain limits the current findings of simulated brain activity with the Ising model. Further research on fiber-tract connectivity in brain networks will improve simulations of self-organized criticality and metastability of functional brain activity. This will lead to a better understanding of complex brain phenomena such as cognition or generating consciousness. While simulating the Ising model provided the opportunity to characterize the structure-function relationship in emergence of complicated brain organizations, the research on graph theoretical approaches (see next section) could offer a better insight to understand the information traffic, and the integration properties of the network.

## 4. A Brief Review of Graph Theory

### 4.1. Current Progress on Graph Theory

Initial approaches of the network structures based on graph theory have developed a growing interest among the researchers involved in investigating the neuronal systems of the brain. Graph theory is providing a simplified and more generalized approach to studying the complex neuronal structures (e.g., Brain network) in neuroscience [82]. Furthermore, it has been proposed that the structure of the global brain network enhances the interaction between the segregation and integration of functionally specialized areas in the brain [83]. Even though the functional networks are restrained by the limitations of the structural connections, context-sensitive integration during cognition tasks necessarily requires a divergence between structural and functional networks. This essential idea is well explained by the “small-world” networks in graph theory which deals with highly clustered, yet globally interconnected networks [84]. The higher the clustering, the greater is the ability of being connected with groups of neurons in the brain network, resulting in network hubs. Thus, it describes the strong functional organization of the brain network and it is also evident in networks which have been extracted in resting state fMRI [84]. In addition, such networks have been described in cortical structure [42, 85] and in EEG and MEG (magnetoencephalogram) studies. Therefore, it is important to compare the parallel behaviour of the organization of functional and structural neuronal anatomy in the brain, and the complex networks of graph theory.

### 4.2. Fundamentals of Graph Theory

Graph theory is an outstanding basis from which to study the functional and anatomical connections in the brain. A graph related to the brain network is a model of the neurons or group of neurons in patches of cortex (nodes/vertices in graph theoretical nomenclature) which are interconnected by a set of edges. The edges represent functional or structural connections between cortical and subcortical regional nodes based on analysis of human neuroimaging data [39]. Nodes in large scale brain networks usually represent brain regions, whereas the edges represent anatomical, effective or functional connections. In a graph, the number of connections a node has is called degree *k* [86]. The distribution of the degree *P*(*k*) gives the information about the fraction of nodes having *k* number of edges and is, therefore, the probability distribution of the degree over the whole network. Clustering coefficient of a graph is another commonly used characteristic which gives the ratio of the number of existing connections to the number of all possible connections [44, 87], whereas the characteristic path length is the average of the shortest path lengths between the nodes. In addition, the global efficiency gives the inverse of the harmonic mean of the minimum path length, between each pair of nodes [88–90] and it indicates the amount of traffic that the network can handle. The local efficiency indicates a measure of the fault tolerance of the network [88] which gives information about handling traffic by each node in the network. In another perspective, efficiency is a useful network measure, which can be used to distinguish between highly active networks or otherwise. Moreover, the strength of divisions of a network in clusters is given by the modularity [91]. High modularity could establish strong connectivity of nodes within clusters and sparse connectivity between nodes of different clusters in the network. The complex networks, which are fundamentally characterized by these network metrics, are complex not only by the means of the size of the network, but also due to the interaction architecture and dynamics of the network [92].

The networks have been classified, according to their topology, under three categories designated random network, small-world network, and scale-free network. Random graphs can be constructed by assigning connections between pairs of nodes with uniform probability. For most of the complex network systems, a random network is a poor estimate. The probability distribution of the degree of a random graph follows a normal distribution as the connections are made randomly between the nodes [92]. The clustering coefficient of random graphs is much smaller compared to that of scale-free and small-world networks. On the other hand, the small-world network is highly clustered yet comprises a smaller characteristic path length compared to random networks [44]. Small-world networks maintain a balance between network segregation and integration, providing a high global and local efficiency of information transfer between nodes of a network [39]. In scale free networks, the nodes are connected in a way that there are few nodes which have very many connections, and many nodes which have few connections [92] implying low efficiency. This is in contrast to with small-world networks where the efficiency is comparatively high, supporting high information transfer between the nodes. On the other hand, the low efficiency of the scale free networks will give the impression of mostly unconnected network structures as well [93]. Although this characterization among the network structures is common, in relation to brain dynamics, a brain network can also be characterized with respect to its regional anatomical connectivity.

### 4.3. Types of Topological Connectivity

Topological connectivity of the brain may characterize different features of dynamic organization. This organization can be expressed by weighted or unweighted graphs. In weighted graphs, nodes represent the regions of interest and edges encode the strength of their correlation (functional) or the density of the fibers connecting them (anatomical) [94], while in unweighted graphs edges only represent the presence of connectivity exceeding a specific threshold. However, this binarization does not provide any information on important differences between weak and strong connections. For specific metrics such as the characteristic path length, the strength of connection is critical for interpretation since it determines the functional distance of connectivity, which is important to characterize long-distance shortcuts. By comparing functional and anatomical connectivity, a broader understanding of the way the brain functions with respect to its structural connectivity can be gained.

#### 4.3.1. Anatomical Connectivity

Anatomical/Structural connectivity between cortical regions of the brain is represented by the connections of axonal fibers. It ranges from inter-neuronal connectivity to inter-regional connectivity in the brain [10]. In MRI analysis, the anatomical connectivity is being tracked using DTI data. Graph theory offers a quantitative description of the anatomical patterns by producing a graph for the anatomical network of the brain. Mapping the anatomical connections of the human brain using graph theory has revealed small-world attributes with local clusters of brain regions [10]. This pattern of finding indicates that the structural organization of the brain demonstrates the most efficient type of network.

#### 4.3.2. Functional Connectivity

Although studying the anatomical connections of the brain allows us to understand the basic structural connectivity throughout the brain, investigating the functional connectivity provides us the knowledge of how this structural architecture relates to brain function. However, functional connectivity is based on statistical computations representing only correlations between nodes exceeding a specific threshold [31, 94]. In fMRI analysis, functional connectivity is analyzed using the BOLD signals. Therefore, functional connectivity emphasizes highly functionally correlated regions. As a result of that, the network can be presented as a fully connected graph among functionally active regions [94]. Thus, measure of efficiency of a network is always a relative quantity dependent on the graphical analysis itself and the choice of threshold.

#### 4.3.3. Effective Connectivity

Effective connectivity refers to the effect that one neural system or element influences another neural system or element [10, 31]. Using effective connectivity, the causal interactions between the elements of the network can be better understood. In this case, a directed graph can be generated to represent the effective connectivity between multiple regions of the brain network [10]. Applying directed graphs is a more sophisticated approach since it also provides information about the direction of connectivity. However, this adds statistically relevant issues when computing fMRI data because temporal aspects of interaction have to be considered which are problematic when measuring the BOLD signal (for further discussion, see [95–98]).

### 4.4. Neurobiological Implications of Graph Theory

#### 4.4.1. The Network Properties in Ising Model and the Brain Network

Studying network properties in graph theory allows a comparison of networks using Ising model data and empirical fMRI data. An important measure in this comparison is the distribution of the degree for the two types of data sets, along with the distribution of the correlation. This will provide the basis to compare the functional behaviour of the brain network in resting state with the critical phenomena of the Ising model.

**Figure 5:**
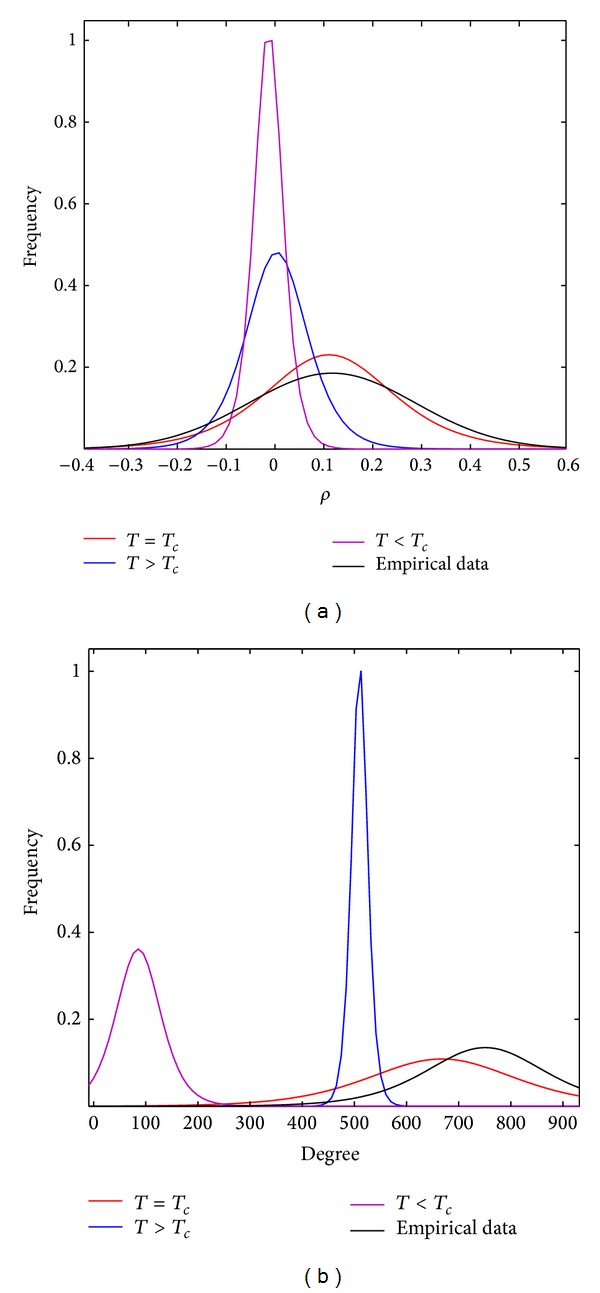
(a) shows the correlation values (*ρ*) distribution for three different simulations of the 2D Ising model at *T* < *T*_*c*_, *T* = *T*_*c*_, and *T* > *T*_*c*_ together with the empirical data from resting state fMRI. (b) shows the degree distribution of the graphs obtained from the corresponding correlation matrices after setting a threshold equal to zero.

After calculating, for each given temperature, configurations of the 2D Ising model at a chosen number of time points, a correlation matrix can be extracted and compared with the correlation of the empirical data from the resting brain (Figure 5). In Figure 5(a), the correlation distribution of empirical and simulated data is showing an important similarity when the Ising model is simulated at the critical temperature.  Figure 5(b) represents the degree distribution for the graphs generated by the corresponding correlation matrices after setting a threshold to zero (using the Brain Connectivity Toolbox [86]). Beyond the critical temperature, the correlation of the simulated data tends to go to zero. At the critical temperature, the 2D Ising model, which does not assess the quality or quantity of information processing, is reflecting the distribution of correlation values of experimental data relevant to brain dynamics [28]. In addition, the distribution of the degree for the 2D Ising model at the critical temperature follows a similar behavior as the experimental data. At temperatures below the critical temperature, the average degree is found at a smaller value compared to that of the resting fMRI degree distribution, which implies lower functional connectivity of the nodes in the Ising model.

The brain network follows small-world behaviour with higher efficiency and a higher clustering coefficient with respect to random or scale-free networks [84]. Moreover, it has shorter average path lengths and most of the connections are made among the neighbouring nodes, while few long-range connections are made in order to create short cuts. In the case of the Ising model, the variation of the degree distribution along with the temperature, allows extraction of valuable information about the network. Below the critical temperature the degree of connectivity of most nodes lies around 10% of the highest possible degree while at higher temperatures it is around 50% of the highest possible degree. These two cases, sub- and supercritical regimes, show a low efficiency in the network, suggesting that the critical behavior for the Ising model is predicting the highest information transfer in accordance with the resting brain data. As the brain network shows small-world network behaviour with lower characteristic path length and higher clustering coefficients in controls, under pathological or pharmacological conditions these properties could be altered depending on the structural or functional modulations of the network.

To understand the structural organization of the brain network and its functional interaction, research has focused on brain alterations. Patients with severe brain injury are especially interesting to investigate, as alterations in structural connectivity can be isolated and compared to loss of function to further explore the relationship between structural and functional connectivity. Moreover, severe brain injury is characterized by a large-scale network disconnection which is the prime mechanism for the underlying cognitive impairment [99]. A prominent impairment in patients with severe brain injury is altered consciousness. In severe chronic states this is defined as disorders of consciousness (DOC) and comprises coma, vegetative state/unresponsive wakeful syndrome (VS/UWS), minimally consciousness states (MCS), and locked-in syndrome (LIS) [100]. In the presence of severe brain injury, the structure of the brain network can be crucially affected. This may lead to a disruption in the functional connectivity of the brain network, which can be captured by their graphical properties [101]. It has been observed by Crone et al. [91] that the functional brain networks of patients with DOC demonstrate a higher clustering coefficient compared to random networks, but a similar characteristic path length, which verifies the small-world attributes in both healthy controls as well as patients with DOC. In comparison to healthy subjects though, the patients show reduced modularity at the global level that implies a shift in the ratio of the connection density within and between clusters. This indicates a disturbance in the optimal balance between integration and segregation.

Altered states of consciousness can also be observed without changes in the structural connectivity as induced, for example, by the anesthetic propofol. In anesthesia-induced loss of consciousness, functional connectivity is disturbed while the structural connectivity is preserved. In respect to graph theory, this can be interpreted as a decrement of the number of functional connections. Graph theoretical analyses revealed significant changes in the distribution of degree and local functional organizations of brain networks during propofol-induced loss of consciousness [102] Recently, Monti et al. investigated the increase in clustering and characteristic path length and the decrease in efficiency of global information flow in propofol-induced unconscious brain networks, compared to wakefulness, mild-sedation, and recovery states of the brain [103]. In their studies, loss of consciousness in the sedation state was characterized as the result of increasing the segregation effect in functional brain organizations.

## 5. Conclusions and Discussion

Throughout this paper, we have reviewed the structure-function relationship in the brain network with recent ongoing analyses, focusing on the Ising model and graph theory. The Ising model together with graph theory proved to be effective approaches to studying brain dynamics. In particular, the Ising model is involved in characterizing the emergent properties of functional network organizations at the critical temperature and the changes in organization when temperature is departing from its critical value. Three significant temperature values are taken into account as the critical, subcritical, and supercritical temperatures. Much of the earlier efforts have compared brain dynamics with the behaviour of self-organized criticality at the critical point of the Ising model. However, the recent finding of characterising brain dynamics in the Griffith phase has started diminishing the hallmark of self-organized criticality in brain networks, unless the network becomes highly efficient and optimized [104]. Their analyses provide the opportunity to look into the behaviour of functional networks based on Ising model simulation in subcritical and supercritical temperature regions in order to understand the macroscopic brain mechanisms. On the other hand, graph theory has been providing another platform to characterize the structural and functional connectivity of the brain. Underpinning results of graph theoretical metrics reveal that the brain network follows a small-world behaviour with a high efficiency and low wiring cost [54, 84]. Furthermore, graph theoretical measures provide additional understanding about the information transfer among the nodes of the Ising model at the critical temperature and in the sub- and supercritical regimes.

The brain is one of the most complex networks in nature due to its sophisticated structure-function relationships. Understanding the optimized information processing and transfer in its cortical networks is the prime focus of much current neuroscience research. With recent advancements in neuroimaging techniques like fMRI (with high spatial resolution), EEG, and MEG (with high temporal resolution), any functional activity based measurements could quantify global correlation patterns in wakeful resting brains [5] or altered states of consciousness as induced by anaesthesia or severe brain injury [33]. Current neuroimaging techniques enable us to explore multiple functional networks within the resting brain with resolution of the order of 10^5^ neurons in a cubic millimeter of neuronal tissue [105]. With this in mind, neuroimaging studies are limited in their characterization of individuals' functionality within any correlated network. However, several of the macroscopic brain phenomena, for example, consciousness, mind, human cognition, global information processing, have recently been investigated in the resting brain with multidimensional analyses of the brain organization in various spatial and temporal scales. With the aid of mean field theory, the functional connectivity of these networks has also been compared with the simulated self-organized criticality of the Ising model in absence/presence of anatomical connectivity.

**Figure 6:**
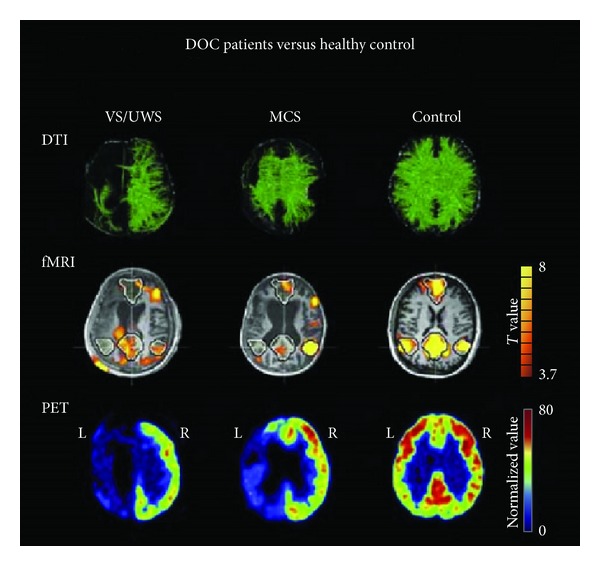
Diffusion tensor imaging, default mode network as extracted from resting state functional magnetic resonance imaging and 18F-fluorodeoxyglucose positron emission tomography in a vegetative patient, a minimally conscious patient, and a healthy control. This figure was modified by Bruno et al. [107].

Nowadays, multimodal neuroimaging is applied to patients with DOC in order to find diagnostic tools [106]. In Figure 6, DTI, fMRI, and FDG-PET are presented for a patient in vegetative state (VS/UWS) and a patient in minimally conscious state (MCS) together with a healthy subject [107]. While the resting-state fMRI and FDG-PET images present a functionally preserved right hemisphere for both patients, DTI shows underlying differences in structural connectivity. In VS and MCS patients, these neuroimaging methods complement each other to provide information of structural and functional connectivity. In recent years, DOC, which could be a result of impaired regulation of arousal and awareness due to connectivity disruptions among different anatomical brain regions [108], have been extensively studied [103, 109, 110]. Their findings highlighted the strong dependency of structure and function in brain networks. Applications of structural and functional neuroimaging, together with computational modeling like the Ising model may allow accessing the spatiotemporal organization of the resting brain and its possible reorganization or disruption in altered states of consciousness.

A recent review also highlighted the fact that structural brain damage after traumatic brain injury (TBI) could disrupt the functional activity of large-scale intrinsic connectivity networks as well as interactions of the damaged structure with neuroinflammation and neurodegeneration as in Alzheimer disease and chronic traumatic encephalopathy [99]. Traumatic focal brain injury may disconnect large-scale brain networks that might result in network dysfunction and cognitive impairment. Their investigations on structural and functional integrity within intrinsic connectivity networks may help to improve diagnosis at the individual network level and clinical treatment in future research. However, the difficulty of accessing long-term human brain data after TBI constrains current studies of DOC, which are mostly treated on the basis of “trial” and “error.” In patients with TBI, diffuse axonal injury may damage structural network connectivity via white matter fibers, which is difficult to investigate through the current tractography technique [111, 112]. This demands the necessity of studying computational models that may help to understand in vivo structure-function relations as well as neuronal intercommunication in large scale brain networks [10].

Graph theory is a useful tool for understanding the organization of brain networks in different spatial and temporal scales. In the healthy brain during rest, the organization within and between RSNs demonstrates small-world features which maximize the information transfer by a relative low level of wiring cost [10]. Together with findings from Ising model simulations explaining the self-organized criticality of brain dynamics, graph theory has opened the door to understand specific properties of organization among these self-organized functional modules [8]. This knowledge can now be used to explore neurobiological mechanisms of the brain network and its alterations in pathological or pharmacological states to better understand how brain phenomena such as cognition or consciousness emerge [1]. This knowledge can then be used to improve innovative biomarkers for the diagnosis and prognosis of disease.
